# A Combined Technology to Protect the Anatomic Integrity of Distal Urethral Sphincter Complex in Radical Prostatectomy Improves Early Urinary Continence Recovery Without Sacrifice of Oncological Outcomes

**DOI:** 10.3389/fonc.2021.711093

**Published:** 2021-08-05

**Authors:** Ao Liu, Yi Gao, Hai Huang, Xiaoqun Yang, Wenhao Lin, Lu Chen, Danfeng Xu

**Affiliations:** ^1^Department of Urinary, Ruijin Hospital, Shanghai Jiaotong University School of Medicine, Shanghai, China; ^2^Department of Pathology, Ruijin Hospital, Shanghai Jiaotong University School of Medicine, Shanghai, China

**Keywords:** radical prostatectomy, urinary incontinence, neurovascular bundle, urinary sphincter, reconstruction, learning curve

## Abstract

**Purpose:**

Our primary aim was to present a combined technique to protect the anatomic integrity of distal urethral sphincter complex (DUSC) during minimally invasive radical prostatectomy (RP) and discuss its impact on urinary continence (UC) recovery. The second aim was to define the learning curve of the combined technique.

**Methods:**

We conducted a non-randomized retrospective study. There were 314 consecutive patients who received RP by the same urologist surgeon with more than 2,000 prior cases in Shanghai Ruijin Hospital between March 2017 and April 2020. Included in this study were 263 patients with clinical T1–T2 stage. We modified a combined RP (Comb-RP) technique including endopelvic fascia no-incising technique, dorsal venous complex (DVC) no-ligation technique, intrafascial dissection technique, and anterior reconstruction technique so as to preserve the anatomic integrity of DUSC. The patients were assigned to two groups: a Comb-RP group and a conventional RP (Conv-RP) group. Continence rates were assessed every 3 months after removal of the catheter. UC was defined as 0 pad per day. Peri-operative variables of the patient including operation time, estimated blood lost (EBL), positive surgical margin (PSM), and postoperative complications were also collected. Scatter-graphs of learning curves were drawn using locally weighted scatterplot smoothing (LOWESS).

**Results:**

RP was accomplished smoothly in all 263 cases. The pad-free UC rates in Conv-RP group and Comb-RP group were 17.3 *vs.* 27.8% (*P* = 0.048) at the removal of the catheter, 35.8 *vs.* 50.0% (*P* = 0.027) at 1 month, 60.5 *vs.* 76.1% (*P* = 0.012) at 3 months, 87.7 *vs.* 96.5% (*P* = 0.022) at 6 months, and 94.7 *vs.* 97.7% (*P* = 0.343) at 12 months. Kaplan–Meier analysis showed significantly higher and faster continence recovery in the Comb-RP group (mean 4.9 *vs*. 2.6 months, Log Rank *P* = 0.001). There was no significant difference in PSM rate between the Comb-RP and Conv-RP group (31.1 *vs*. 31.2%, P =0.986). The learning curves of peri-operative variables, oncological and functional outcomes achieved the lowest point or plateau at the 20th–60th cases.

**Conclusions:**

The anatomic integrity and intact pelvic floor interplay of DUSC is important for its function. Our combined technique was a safe and feasible technique for improving early UC in RP with no significantly increased PSM rate and no significant difference in long-term UC.

## Introduction

Radical prostatectomy (RP) is one of the most important methods for the treatment of localized prostate cancer (PCa). On the premise of ensuring the effect of tumor control, protection of urinary continence (UC) has long been the crux in RP. Various techniques for early UC recovery have emerged in recent years. The early UC recovery rate of the above techniques is reportedly 23–84% (1–8). Among different UC theories, the urethral sphincter has been widely accepted as a significant factor for improving early continence. In addition to the functional-length, the anatomic fixation and integrity of the urethral sphincter are essential to preserving UC after RP (4).

The UC structure is a complex systematic integrity rather a simple combination of individual anatomic structures, which was named as distal urethral sphincter complex (DUSC). In *Campbell-Walsh Urology*, the DUSC refers to the UC structure from the colliculus seminalis extending to the proximal bulbar urethra, including the prostatic urethra, membranous urethra (MU), smooth muscle sphincter of prostatic and membranous urethra, prostatic striated sphincter (PSS), membranous urethra striated sphincter (MUS), periurethral striated sphincter (PUSS), puboprostatic ligaments, and other pelvic connective tissues. The key point of this concept lies in the definition of the complex. It is not a simple physical superposition of multiple anatomic elements; rather, it is an organic integrity in which these anatomic elements link with each other and affect each other. In surgical practice, preservation of the DUSC means maintaining the integrity of these structures and protecting them from being damaged.

As we continually try to improve our technique, we adopt endopelvic fascia no-incising technique, dorsal venous complex (DVC) no-ligation technique, intrafascial dissection technique, and anterior reconstruction technique so as to preserve the anatomic integrity of DUSC as much as possible to ensure its function. We postulate these approaches are key steps to maintain the anatomic integrity and intact pelvic floor interplay of DUSC and can be made smoothly in both robotic and laparoscopy assisted RP.

In the current paper, we described our combined technique and attempted to compare differences between the combined RP (Comb-RP) and the conventional RP (Conv-RP) in terms of the peri-operative data and UC recovery. We also evaluated the effect of surgical experience on peri-operative, functional, and oncological outcomes in the Comb-RP group.

## Patients and Methods

### Study Design and Patient Selection

This was a non-randomized retrospective study. There were 314 patients who received RP by the same urologist surgeon with more than 2,000 prior cases in Ruijin Hospital (Shanghai, China) between March 2017 and April 2020. The inclusion criteria were patients who met the RP indications with clinical T1–2 stages. A pre-surgery prostatic MRI was performed to identify localized PCa without extending through the prostatic capsule or seminal vesicle invasion. Men were scored as 0 according to the International Continence Inquiring Committee’s Questionnaire (ICI-Q-SF). The exclusion criteria were patients who were lost to follow-ups. Using the inclusion and exclusion criteria, a total of 263 cases were finally recruited in this study. The study was approved by the Ethics Committee of Shanghai Ruijin Hospital, and written informed consent was obtained from all patients.

Preoperative baseline data of the patients, operation time, estimated blood loss (EBL), postoperative complications, and 1-, 3-, 6- and 12-month postoperative UC were collected retrospectively. Pad-free UC at catheter removal, 1- and 3-, 6- and 12-month was followed up by telephone interviews that included questions about pad usage and duration of incontinence. UC recovery was defined as no pad. Histopathological analysis was processed according to the recommendations of the American Society of Clinical Pathologists (ASCP) (9).

### Surgical Technique

Patients underwent either RALRP or LRP. Pelvic lymph node dissection (PLND) was performed in patients with risk of LN involvement >5% in the Briganti nomogram (10).

#### Key Steps of the Combined Technique Are Reported Below

**Figure 1:**
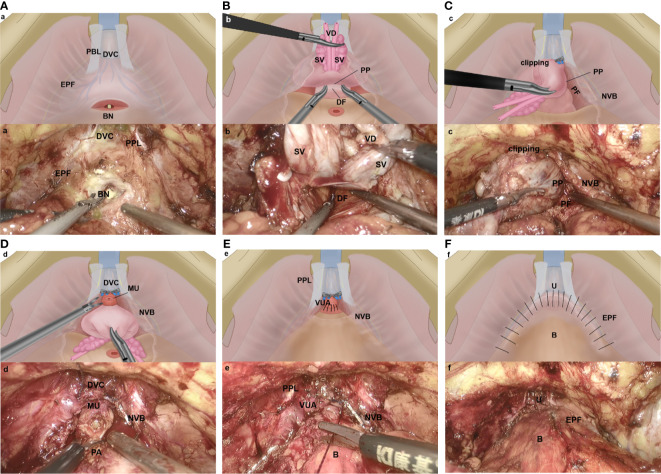
Key steps of the Com-miRP technique **(A–F)**. **(A)** The anterior bladder neck was incised without incising the endopelvic fascia or ligating the dorsal venous complex. EPF, endopelvic fascia; BN, bladder neck; DVC, dorsal vascular complex; PPL, puboprostatic ligaments. **(B)** The seminal vesicle release and vas deferens are transected precisely, and the anterior layer of Denonvilliers fascia is well protected. An incision is made between the posterior capsule of prostate and the anterior layer of Denonvilliers fascia. DF, Denonvillier’s fascia; SV, seminal vesicle; VD, vas deferens; PP, prostatic pseudocapsule. **(C)** By starting at the 5- and 7-o’clock position, we develop an avascular plane between prostatic pseudocapsule and prostate fascia by using the retrograde method. The preservation of the NVB complex is maximized. The DVC is controlled by clipping temporarily in case of bleeding. PP, prostatic pseudocapsule; NVB, neurovascular bundle; PF, prostatic fascial. **(D)** Apical dissection is performed underneath the DVC. The apical dissection is minimized, and the distal urethral sphincter is preserved by retracting the prostate firmly to the head of the patient. The urethra is then transected sharply 5 mm distal to the apical prostate. PA, prostatic apex; NVB, neurovascular bundle; DVC, dorsal vascular complex; MU, membranous urethral. **(E)** Vesicourethral anastomosis is performed with the technology described by Mani Menon et al. (9) PPL, puboprostatic ligaments; VUA, vesicourethral anastomosis; NVB, neurovascular bundle; B, bladder. **(F)** The puboprostatic ligaments, parietal endopelvic fascia, and anterior detrusor apron are reconstructed on both sides to support the anastomosis. U, urethra; B, bladder; EPF, endopelvic fascia.

Patients underwent a transperitoneal six-port robotic surgery or traditional LRP. Robotic/laparoscopy set-up and port placement followed the techniques described by previous studies (11, 12). Some steps and anatomical landmarks of our modified technique are illustrated in **Figure 1**.

##### Bladder Neck Transection

The assistant pulled the Foley catheter back and forth; this simple maneuver aided in the identification of the bladder neck. We approach the bladder neck directly without incising the endopelvic fascia or ligating the dorsal venous complex (DVC), (**Figure 1A**).

##### Preservation of the DVC

We transected the anterior bladder neck without ligating the DVC, which was followed by posterior bladder neck dissection and posterior plane dissection between the prostate and the rectum. This plane was incised precisely; after seminal vesicle release and vas deferens transection, both the vasa and seminal vesicle were then grasped, the posterior prostate was lifted, and the anterior layer of Denonvilliers fascia was well protected. An incision was made between the posterior capsule of prostate and the anterior layer of Denonvilliers fascia, (**Figure 1B**).

##### Intrafascial Prostate Dissection

We avoided incising the prostatic fascia anteriorly, where the fascia was fused with the puboprostatic ligament and covered the dorsal venous plexus. The nerve-sparing technique was performed by starting at the 5- and 7-o’clock position of the posterolateral region; developing an avascular plane between the prostatic pseudocapsule and the prostate fascia; continuing with blunt and cold dissection toward the anterior and distal surface of the prostate, following the intrafascial plane by using the retrograde method; maximizing the preservation of the neurovascular bundle (NVB) complex until reaching the prostatic anterior fibromuscular stroma, (**Figure 1C**). When performed properly, curtains of periprostatic tissue hang from the pubourethral ligament.

##### Apical Dissection Technique and Control Of DVC Bleeding

Apical dissection was performed underneath the DVC, avoiding injury to the anterior vascular structures. After the prostatic fascia was dissected off the prostatic apex, we minimized the apical dissection and preserved the distal urethral sphincter by retracting the prostate firmly to the head of the patient. The urethra was then transected sharply 5 mm distal to the apical prostate, (**Figure 1D**). The DVC was controlled by temporary titanium clipping or selective stitch in case of bleeding, avoiding a deep stitch of the DVC and circumjacent musculofascial tissue. We also tried to protect the cavernosal nerves, which were close to the urethra and were vulnerable to thermal or traction trauma.

##### Urethrovesical Anastomosis and Anterior Reconstruction

A running 2-0 suture (Prolene; Ethicon) was used for the urethrovesical anastomosis, (**Figure 1E**). We used the technique described by Mani Menon (9). Once the integrity of the anastomosis was identified, the puboprostatic ligaments, parietal endopelvic fascia, and anterior detrusor apron were reconstructed on both sides to support the anastomosis. After the anterior reconstruction was completed, the former clippings which were used to control DVC were removed simultaneously, (**Figure 1F**).

Comparatively speaking, Conv-RP was performed in accordance with the NHS routine practice. The endopelvic fascia was incised. The DVC was processed with distal bunching and cut off with an electrosurgical scalpel. As a result, bilateral puboprostatic ligaments were not preserved in Conv-RP group.

Both in the Conv-RP group and Comb-RP group, the drain was removed on days 2–3 postoperatively, and patients were discharged with an indwelling Foley catheter. The catheter was removed on day 14 after surgery.

### Statistical Analysis

SPSS version 22.0 (IBM Corp, Armonk, NY, USA) was used for statistical analysis. Normally distributed continuous variables were reported as means with standard deviations (SDs) and tested by Student’s t-test. Non-normally distributed continuous variables were reported as medians with interquartile range (IQR) and tested by Mann–Whitney U test. For categorical variables, counts and percentages were calculated. Categorical variables in two surgical groups were tested by Chi-square test and Fisher’s exact tests, as appropriate. Kaplan–Meier analyses were used to compare the times to pad-free continence between the Comb-RP and Conv-RP groups. Scatter-graphs of peri-operative, functional, and oncological outcomes were drawn using locally weighted scatterplot smoothing (LOWESS), and a plateau phase was estimated. A two-tailed test with p <0.05 was considered statistically significant.

## Results

A total of 263 RPs were performed, including 173 Conv-RPs and 90 Comb-RPs. The median follow-up time was 21 months. There were fewer patients who underwent robotic surgery in Comb-RP than in Conv-RP. There were no significant differences in age, BMI, American Society of Anesthesiologists (ASA) classification, PSA level, lower urinary tract symptom (LUTS), and prostate volume between the two groups (**Table 1**).

**Table 1:** Baseline parameters of patients undergoing Conv-RP or Comb-RP.

Characteristics	Conv-RP(n = 173)	Comb-RP(n = 90)	*P*
Age, years (mean ± SD)	68.4 ± 7.0	67.7 ± 6.9	0.497^a^
BMI, kg/m^2^ (mean ± SD)	24.6 ± 2.9	24.2 ± 2.8	0.370^a^
ASA, n (%)			0.060^d^
1	3 (1.7)	3 (3.3)	
2	127 (73.4)	75 (83.3)	
3	43 (24.9)	12 (13.3)	
Median PSA ng/ml (IQR)	10.8 (7.5–16.1)	10.8 (7.5–15.4)	0.678^b^
LUTS, n (%)	49 (28.3)	32 (35.6)	0.228^c^
Median prostate volume, ml (IQR)	64.0 (50.0–94.5)	64.0 (50.8–90.5)	0.883^b^
Robotic surgery, n (%)	35 (20.2)	9 (10.0)	**0.035** ^**c**^
PLND, (%)	89 (51.4)	38 (42.2)	0.156

a Student’s t-test.

b Mann–Whitney U test.

c Chi-square test.

d Fisher’s exact test.

SD, standard deviations; IQR, interquartile range; LUTS, lower urinary tract symptom; RP, radical prostatectomy; Conv-RP, conventional radical prostatectomy; Comb-RP, combined radical prostatectomy; PSA, prostate specific antigen; ASA, American Society of Anesthesiologists; BMI, body mass index; PLND, pelvic lymph node dissection. Bold values indicate significant p-values.

### Intra-Operative and Oncological Outcomes

As shown in **Table 2**, the length of operation time in Conv-RP group was longer than that in Comb-RP group (125 *vs*. 110 min, P = 0.011). The distribution of pathological T (pT) stage in Conv-RP and Comb-RP was slightly different, and patients in Conv-RP group tended to have early pT stage. There were no significant differences in estimated blood loss (EBL), International Society of Urological Pathology (ISUP) group, positive surgical margin (PSM), and postoperative complication between the two groups. Prostate margin status was divided into four groups: negative margin, apical PSM, non-apical PSM, and multiple PSM (**Table 3**): 8 (4.6%) patients in Conv-RP group *vs.* 2 (2.2%) in Comb-RP group had positive apical margin, 33 (19.1%) *vs.* 17 (18.9) patients had a positive margin in non-apical site, 13 (7.5%) *vs.* 9 (10.0%) patients had positive margins in multiple site, and 119 (68.8%) *vs.* 62 (68.9%) patients had negative surgical margin. Chi-square test showed no statistical significance between the two groups in all margin status groups.

**Table 2:** Perioperative and pathological parameters of patients undergoing Conv-RP or Comb-RP.

Characteristics	Conv-RP (n = 173)	Comb-RP (n = 90)	*P*
Median operation time in minutes (IQR)	125 (100–145)	110 (95–135)	**0.011** ^**b**^
Median EBL in ml (IQR)	100 (50–200)	100 (50–150)	0.105^b^
pT stage; n (%)			**0.031** ^**d**^
≤pT2c	107 (61.9)	69 (76.7)	
pT3	64 (37.0)	20 (22.2)	
pT4	2 (1.2)	1 (1.1)	
ISUP group; n (%)			0.979^d^
1	16 (9.2)	10 (11.1)	
2	82 (47.4)	44 (48.9)	
3	46 (26.6)	23 (25.6)	
4	7 (4.1)	3 (3.3)	
5	22 (12.8)	10 (11.1)	
PSM; n (%)			0.986^c^
Yes	54 (31.2)	28 (31.1)	
No	119 (68.8)	62 (68.9)	
Postoperative complication; n (%)	3 (1.7)	1 (1.1)	1.000^d^

b Mann–Whitney test.

c Chi-square test.

d Fisher’s exact test.

IQR, interquartile range; RP, radical prostatectomy; Comb-RP, combined radical prostatectomy; Conv-RP, conventional radical prostatectomy; ISUP, International Society of Urological Pathology; PSM, positive surgical margin; EBL, estimated blood loss. Bold values indicate significant p-values.

**Table 3:** Margin details of patients undergoing Conv-RP or Comb-RP.

Margin details	Conv-RP (n = 173)	Comb-RP (n = 90)	*P*
Negative margin; n (%)	119 (68.8)	62 (68.9)	0.986^c^
Apical PSM; n (%)	8 (4.6)	2 (2.2)	0.984^c^
Non-apical PSM; n (%)	33 (19.1)	17 (18.9)	0.971^c^
Multiple PSM; n (%)	13 (7.5)	9 (10.0)	0.490^c^

c Chi-square test.

Comb-RP, combined radical prostatectomy; Conv-RP, conventional radical prostatectomy; PSM, positive surgical margin.

### Functional Outcomes

**Figure 2:**
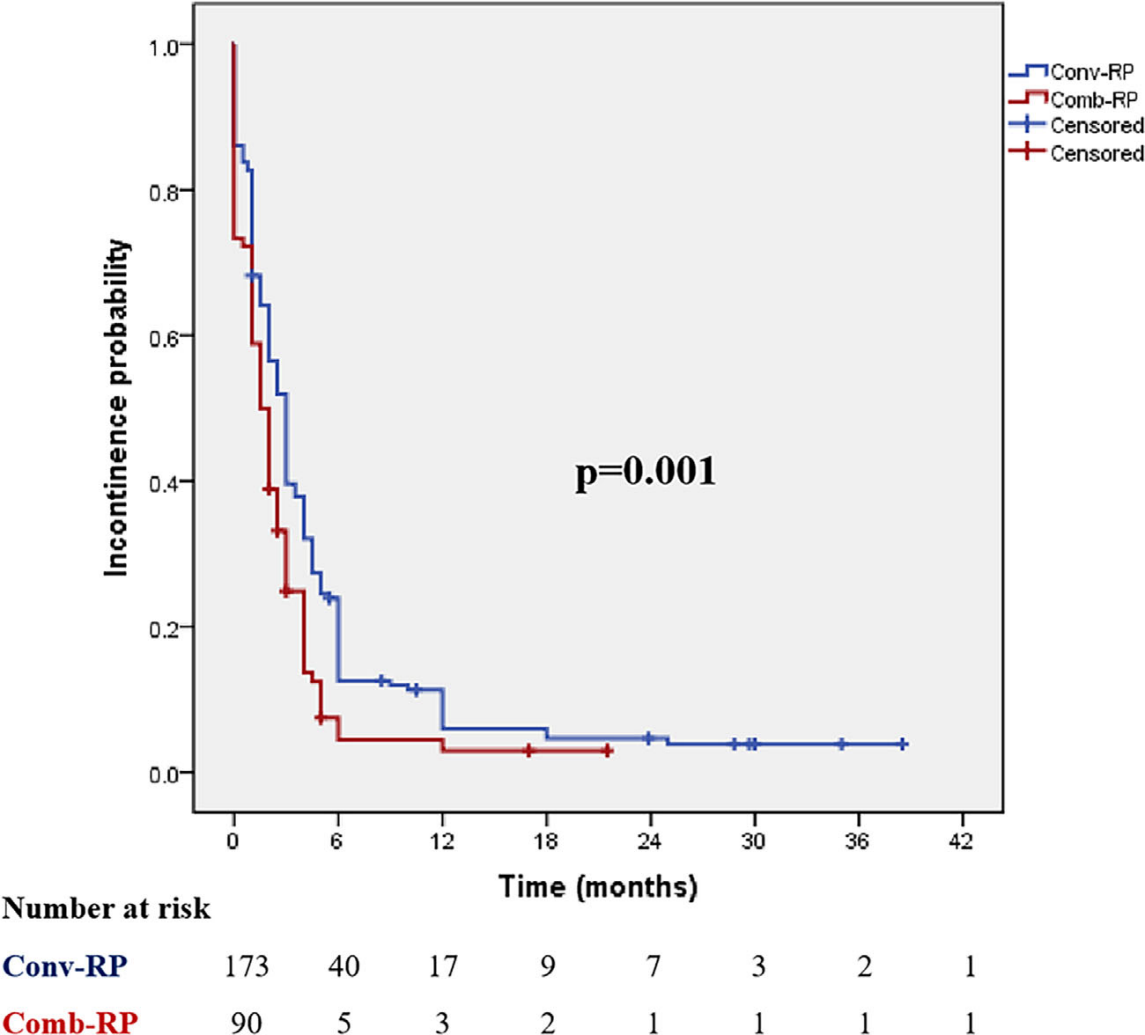
Cumulative probability of incontinence after radical prostatectomy by study groups (Comb-RP and conv-RP). Conv-RP, conventional radical prostatectomy; Comb-RP, combined radical prostatectomy. Log Rank p = 0.001.

The results of comparison of postoperative UC between Conv-RP and Comb-RP were shown in **Table 4**: instant UC: 17.3 *vs.* 27.8% (*P* = 0.048); 1-month pad-free UC: 35.8 *vs.* 50.0% (*P* = 0.027); 3-month pad-free UC: 60.5 *vs.* 76.1% (*P* = 0.012); 6-month pad-free UC: 87.7 *vs.* 96.5% (*P* = 0.022); 12-month pad-free UC: 94.7 *vs.* 97.7% (*P* = 0.343). Kaplan–Meier analysis showed significantly higher and faster continence recovery in the Comb-RP group (Log Rank *P* = 0.001; **Figure 2**). The mean time to achieve pad-free continence was 4.9 months in the conventional group and 2.6 months in the combined RP group.

**Table 4:** Continence outcomes of patients undergoing Conv-RP or Comb-RP.

Continence outcomes	Conv-RP (n = 173)	Comb-RP (n = 90)	*P*
Instant UC; n (%)	30 (17.3)	25 (27.8)	0.048^b^
UC at 1 month; n (%)	62 (35.8)	45 (50.0)	0.027^b^
UC at 3 months; n (%)	104 (60.5)	67 (76.1)	0.012^b^
UC at 6 months; n (%)	150 (87.7)	83 (96.5)	0.022^b^
UC at 12 months; n (%)	160 (94.7)	84 (97.7)	0.343^c^

b Chi-square test.

c Fisher’s exact test.

RP, radical prostatectomy; Comb-RP, combined radical prostatectomy; Conv-RP, conventional radical prostatectomy; UC, urinary continence. Bold values indicate significant p-values.

### Learning Curve Analysis

The learning curve for operation time decreased as the cases increased, reaching the lowest point at the 50th case (**Supplement Figure S1**). The learning cure for EBL after surgery showed a pattern similar to operation time. The lowest point of both was attained at the 50th case (**Supplement Figure S2**). Oncological outcome was reported as PSM rate, which reached a plateau at the 60th case (**Supplement Figure S3**). In terms of the functional outcomes, the learning curve for instant, 1-, 3-, and 12-month UC reached a plateau at the 30th, 50th, 40th, 20th cases, respectively (**Supplement Figure 4**).

## Discussion

Although RALRP has improved postoperative UC remarkably as compared with conventional LRP (13, 14), urinary incontinence remains the main postoperative complication affecting the quality of life of such patients (15). In the present study, we introduced a new combined technique which helped early UC recovery but showed no significant difference in long-term UC as compared with conventional RP. Our result demonstrated that the Comb-RP technique was safe and feasible as there were no significant differences in the operation time, EBL, PSM rate, and postoperative complications between the Comb-RP group and Conv-RP group. The learning curve also indicated all peri-operative, functional, and oncological outcomes reached the lowest point or plateau in an early period.

Using the combined technique, the instant, 1-, 3-, and 6-month UC recovery rates improved significantly as compared with Conv-RP. The possible reasons for such improvement can be summarized in the following points.

1. Preservation of the neurovascular structure by using the DVC no-ligation technique, the NVB dissociation and preservation technique without thermal and mechanical damage (8), and the technique to minimize apical dissection (16) so that the vessel and nerve in the apex of prostate can be maximally protected.
2. Preservation of the muscle, fascia, and ligament supporting structures by using the bladder neck-sparing technique (17), membranous urethral length preservation technique (18), and the minimal apical dissection technique (16) to maximally preserve the muscular UC tissue in the bladder neck, the urethra rhabdosphincter in the apex, and the levator ani muscle around the apex, which is considered as important support structure.
3. Anterior reconstruction is important. We sutured the muscular fibers of the bladder neck to the periurethral tissue between the DVC and the anastomosed urethra and realigned the bladder to the pelvic sidewall with the aim to restore the periurethral support and vesicourethral angle.

PSM is recognized as an important factor in biochemical recurrence and disease progression; the PSM rate reported in previous studies is 8.6–33.2% (12, 19–21). The PSM rate in our study was similar with that in previous studies. On the one hand, PSM may be due to the intrafascial resection technique itself (22). On the other hand, PSM may be explained by the heterogenicity of the included patients as the high proportions of PCa patients with a pT3a or higher stage. Previous studies suggested that the location, length, and number of PSM are also important (20). The apical PSM represents the most common site after RP, especially miRP (21). However, positive margin at the apex showed less influence than other peri-operative variables, like maximum positive biopsy cores on biochemical recurrence (19). Finding from our analysis confirmed that the non-apical site is the most frequent location of PSM; differences of PSM location likely reflect variations in surgical technique. However, we did not find any significant difference in terms of PSM between patients in Comb-RP group and those in Conv-RP group.

Understanding the learning curve of a new surgical technique is very important for surgical education. To date, some studies have reported the learning curves of LRP and RALRP. An early study reported the safety and efficacy of Retzius-sparing RALRP, and there was a linear relationship between surgery experience and imitated UC in the first 50 cases, without reaching a plateau (23). Some authors asserted that with >100 cases, the plateau on early continence is achievable (24). Regarding PSMs, the evidence available is controversial: some researchers did not find improvement of the PSM rate during the learning curve (23, 25), while others, in a single surgeon series, found an overall reduction of positive margins with the amount of experience (26). In an early series about traditional RARP, Patel and colleagues (27) showed that operative times and EBL are reduced with the experience of the surgeon. It has been demonstrated that longer surgical experience also decreases complications. In this study, while PSM rate was stable as the cases increased, operation time, EBL and LOS after surgery gradually increased after the lowest point. Possible explanations are as follows: first, there was a tendency to perform more difficult cases while the experience increased. Second, we tried new technique in patients with intermediate to high-risk PCa. Finally, more robotic surgeries were performed in recent years, and the surgeon needed more time to transfer from a laparoscopic environment to using a robotic interface.

Porpiglia et al. (28) reported 5-year functional and oncological outcomes of their previously published prospective randomized study comparing robot-assisted radical prostatectomy (RARP) and laparoscopic radical prostatectomy (LRP). Their results suggested that the probability of achieving continence [odds ratio (OR) 2.47, p < 0.021] and potency (OR 2.35, p < 0.028) over time was more than doubled for the RARP compared to the LRP group. In our study, 20.2% of patients in Conv-RP group underwent RARP, while only 10.0% in Comb-RP group selected robotic surgery. However, the Comb-RP group finally achieved better functional outcomes with less proportion of patients who underwent robotic surgery, which fully suggested our Com-RP technology has a significant positive influence on UC.

The main limitation of our data arises from its retrospective design. The study also has some other limitations. First, it is necessary to conduct follow-up study to trace the long-term oncologic outcome to ascertain the impact of this technique on tumor control. Second, it is still necessary to obtain data from randomized prospective studies to support our conclusions. Third, this technique should be used with caution in high-risk PCa patients according to the concrete situation. It is relatively safe in tumor control of low- and intermediate-risk PCa patients in the context of high-quality research data currently available (29, 30).

## Conclusion

This study was based on the DUSC anatomic theory, and the results have demonstrated that the new technique is a relatively safe, effective, and reliable technique for tumor control and early UC in RP. Additionally, learning curves of peri-operative outcomes including operation time and EBL achieved the lowest point during a short learning process. UC and PSM rates also attained a plateau at a similar period of time.

## Data Availability Statement

The raw data supporting the conclusions of this article will be made available by the authors, without undue reservation.

## Ethics Statement

The studies involving human participants were reviewed and approved by the Ethics Review Board of Ruijin Hospital. The patients/participants provided their written informed consent to participate in this study.

## Author Contributions

All authors contributed to the article and approved the submitted version. DX, LC, and AL designed the research study. XY, YG, and HH contributed essential reagents or tools. AL and WL analyzed the data. AL and LC wrote the paper.

## Funding

This study was funded by National Natural Science Foundation of China (No. 81972405); Shanghai Committee of Science and Technology, China (No. 18411960100; No.20Y11904700).

## Conflict of Interest

The authors declare that the research was conducted in the absence of any commercial or financial relationships that could be construed as a potential conflict of interest.

## Publisher’s Note

All claims expressed in this article are solely those of the authors and do not necessarily represent those of their affiliated organizations, or those of the publisher, the editors and the reviewers. Any product that may be evaluated in this article, or claim that may be made by its manufacturer, is not guaranteed or endorsed by the publisher.
