# Supplementary material for: A Combined Technology to Protect the Anatomic Integrity of Distal Urethral Sphincter Complex in Radical Prostatectomy Improves Early Urinary Continence Recovery Without Sacrifice of Oncological Outcomes
Source: Front Oncol. 2021 Aug 5;11:711093. doi: 10.3389/fonc.2021.711093 (PMC8374866; doi:10.3389/fonc.2021.711093)
Supplement: Supplementary file 1 [file DataSheet_1.docx]

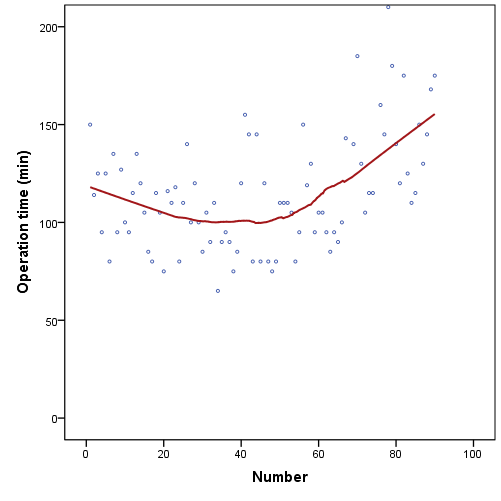


**Supplement Figure1. The learning cure for operation time.**


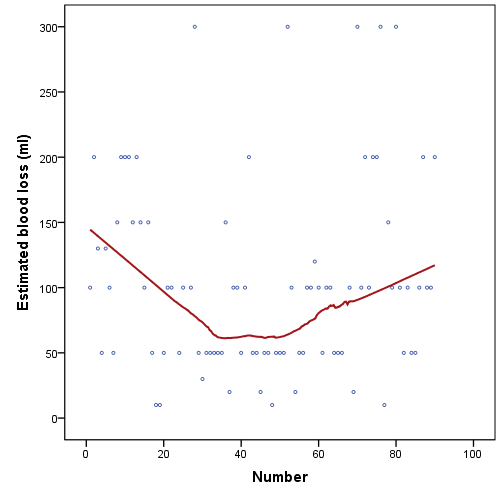


**Supplement Figure2. The learning cure for estimated blood loss.**


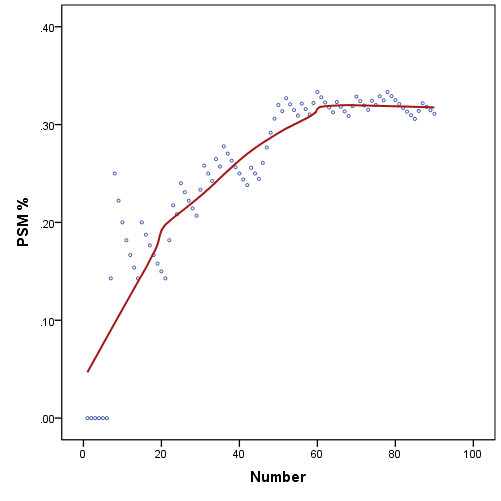


**Supplement Fgure3. The learning cure for positive surgical margin (PSM) rates (cumulative sum).**

**
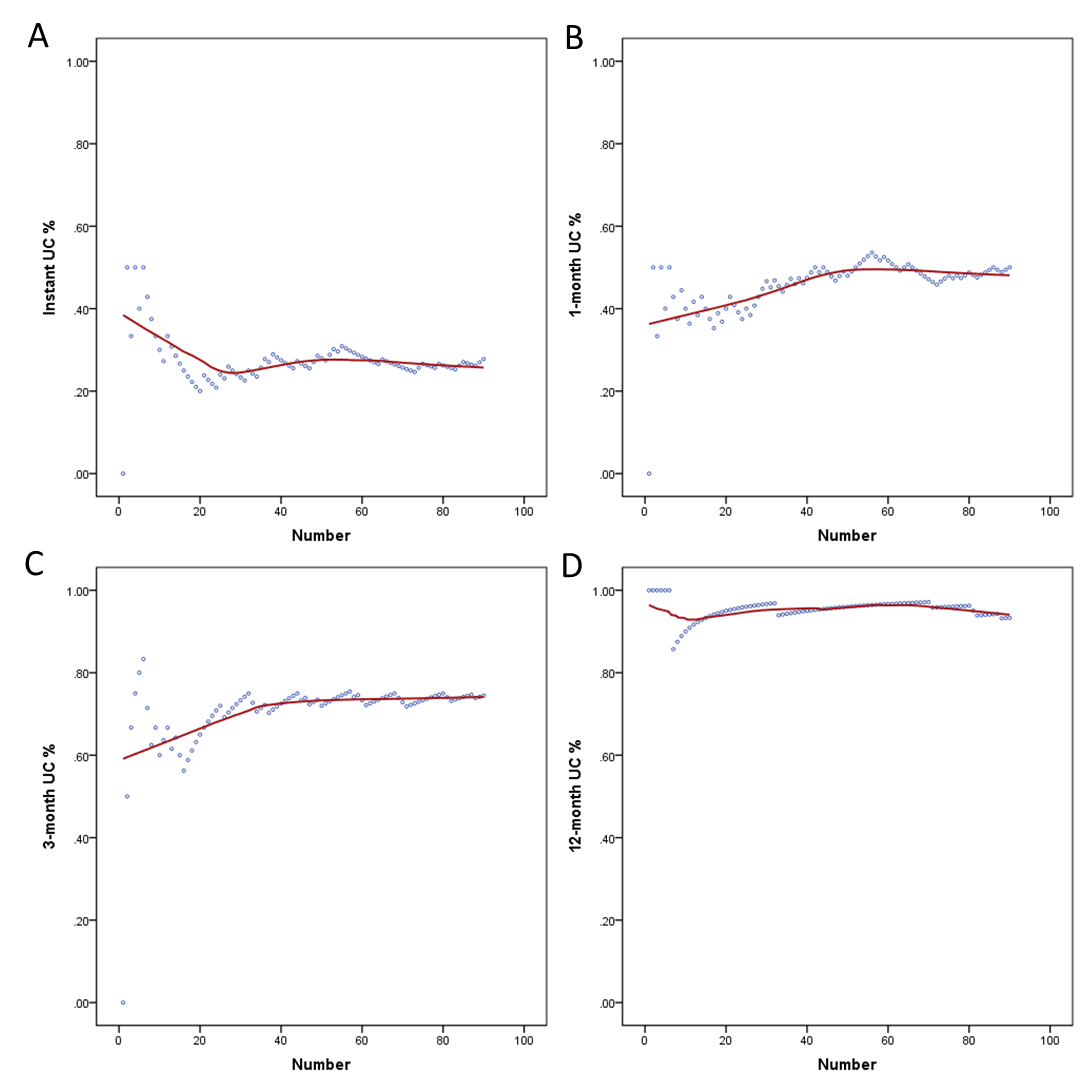
**

**Supplement Figure4.The learning curve for the functional outcomes. (A) Cumulative sum probability of instant urinary continence (UC) , (B) Cumulative sum probability of 1-month UC, (c) Cumulative sum probability of 3-month UC, and (d) Cumulative sum probability of 12-month UC.**
